# Can Mixed-Species Groups Reduce Individual Parasite Load? A Field Test with Two Closely Related Poeciliid Fishes (*Poecilia reticulata* and *Poecilia picta*)

**DOI:** 10.1371/journal.pone.0056789

**Published:** 2013-02-20

**Authors:** Felipe Dargent, Julián Torres-Dowdall, Marilyn E. Scott, Indar Ramnarine, Gregor F. Fussmann

**Affiliations:** 1 Department of Biology, McGill University, Montreal, Quebec, Canada; 2 Department of Biology, Colorado State University, Fort Collins, Colorado, United States of America; 3 Institute of Parasitology, McGill University, Ste-Anne de Bellevue, Quebec, Canada; 4 Department of Life Sciences, University of the West Indies, Saint Augustine, Piarco-Tunapuna, Trinidad and Tobago; University of Jyväskylä, Finland

## Abstract

Predation and parasitism are two of the most important sources of mortality in nature. By forming groups, individuals can gain protection against predators but may increase their risk of being infected with contagious parasites. Animals might resolve this conflict by forming mixed-species groups thereby reducing the costs associated with parasites through a relative decrease in available hosts. We tested this hypothesis in a system with two closely related poeciliid fishes (*Poecilia reticulata* and *Poecilia picta*) and their host-specific monogenean ectoparasites (*Gyrodactylus spp.*) in Trinidad. Fish from three different rivers were sampled from single and mixed-species groups, measured and scanned for *Gyrodactylus*. The presence and abundance of *Gyrodactylus* were lower when fish of both species were part of mixed-species groups relative to single-species groups. This is consistent with the hypothesis that mixed-species groups provide a level of protection against contagious parasites. We discuss the importance of potentially confounding factors such as salinity and individual fish size.

## Introduction

Forming groups is an adaptive strategy seen in many animal species [1]. Individuals that join a group of conspecifics often gain benefits that increase their fitness compared to those that do not join a group; however group formation also involves interactions that are costly to the individual. Two major benefits of group forming behaviour are a decrease in an individual’s probability of being preyed upon and an increase in the probability of acquiring food [2]. A major cost that individuals experience when joining a group is increased contagious parasite transmission [3]. Contagious parasites lack mobile dispersal stages [3], therefore their transmission depends on the number and frequency of contacts between hosts. As group size increases, increased contact between suitable hosts [4] results in higher mean number of contagious parasites per individual and higher percentage of infected individuals [3], [5], [6]. Conversely, mobile parasites need to search or ambush their hosts; as group size increases this behaviour will likely decrease a host individual’s probability of receiving a mobile parasite attack [7], [8].

Individuals also form groups with heterospecifics [1], [4], [9]–[11] when members of more than one species overlap spatially and temporally [1], [9]. Active behavioural choice of at least one participating species is inferred for the formation of these groups as their occurrence is far more frequent than would be expected by chance [9], [12]. Attempts to explain the advantages of forming mixed-species groups focus on anti-predation mechanisms and, less prominently, on foraging advantages [12], [13]. When anti-predator mechanisms are evoked individual benefits derive from the numerical increase in group members; these are essentially the same mechanisms that are at work in single-species groups and cannot explain the particular advantage of a heterospecific association. For example, this is the case for anti-predation mechanisms where the benefit resides in diluting the risk of attack [14] or in reducing exposure to predators [15]. Foraging advantages, in contrast, result from the heterospecific nature of the association. For example, individuals in mixed-species groups can acquire more resources than those in equally sized single-species groups by reducing competition (i.e. interspecific competition is weaker than intraspecific competition [13], [16]).

Contagious host-specific parasitism provides an alternative explanation of why individuals in mixed-species groups would experience higher fitness compared with equally sized single-species groups [4]. When individuals face the combined threat of predation and parasitism, joining a mixed-species group may be an adaptive strategy that, on aggregate, outperforms either of the pure protective strategies. In mixed groups, under certain circumstances (i.e. when not strongly phenotypically dissimilar [17], [18]), individuals can enjoy similar anti-predator protection as in single-species groups (“all individuals count”) but can also decrease their risk of being parasitised due to dilution of the group with individuals of the second species that are not susceptible to infection (“only individuals of the host species count” [4]). This effect has been largely overlooked as an explanation for mixed-species group formation, although increasing evidence of the negative correlation between species diversity and parasite transmission has been accumulating during the last decade (“dilution effect” e.g. [19]–[21] but see [22]).

In this study we compared contagious parasite infections in single- versus mixed-species groups of two closely related poeciliid fishes in the wild. We assumed that parasite transmission and/or parasite intrinsic rate of increase was higher on the co-evolved species-specific host-parasite pairs [23]. Therefore we hypothesised that individuals of each host species would experience lower levels of contagious parasites (presence and abundance) by engaging in mixed-species groups.

### The Study System

To test our hypothesis, we used as model organisms two poeciliid fishes that in their native habitat in Trinidad [24] form mixed-species groups, that are infected by different species of contagious parasites and that face the risk of predation by multiple piscivorous fishes. *Poecilia reticulata* (“Trinidadian guppy”) is found in freshwater whereas *Poecilia picta* (“swamp guppy”) is found across a salinity gradient from freshwater to brackish water [24]. In the zones where their distributions overlap, they form mixed-species groups [25]. These two fish species are parasitised by *Gyrodactylus spp*., a group of viviparous monogenean ectoparasites of the skin and fins that are transmitted mainly through host to host contact [26], [27]. Wild *P. reticulata* are infected with *Gyrodactylus turnbulli* and *G. bullatarudis* [28], whereas *G. pictae* have been found only on wild *P. picta* [29]. Under controlled lab experiments *G. turnbulli* and *G. bullatarudis* can survive on *P. picta* but only for short periods of time and with lower reproductive rates than on *P. reticulata* [30], [31]. There is no field evidence of cross-infection.

## Materials and Methods

### Ethics Statement

This study was carried out in accordance with McGill University’s Animal Care Committee and the Canadian Council on Animal Care in Sciences guidelines. Our protocol was approved by the Macdonald Campus Facility Animal Care Committee of McGill University (AUP #5759). Field sampling also received approval of the Ministry of Agriculture, Land and Marine Resources - Fisheries Division of the Republic of Trinidad and Tobago.

### Materials and Methods

We sampled approximately 20 female and 20 male individuals of each *Poecilia* species present at three types of sites on each of three rivers using butterfly nets (Table 1). Two of the three rivers included a *P. reticulata*-only site, a *P. picta*-only site and two sites where mixed-species groups are formed. In the third river (Nariva), only one mixed-species site was sampled. Each collection site was less than a 10-metre longitudinal section of the river. At mixed-species sites, we observed no signs of spatial segregation among fish, so we consider these sites to be representative of individuals of the two species grouping together. Since salinity is a major environmental variable that drastically changes across the *P. picta* distribution, we measured specific conductivity at each site, as a proxy of salinity (YSI probe model 85–50 FT), and report the direct measurement (specific conductivity). After capture, fish were first placed individually in 18 oz Whirlpak bags to avoid parasite transmission during transport to the lab. The total capture period at single species sites was no longer than 2 hours, and at mixed species sites was no longer than 4 hours. This included the time it took to find fish shoals, to capture a random sample of its members, to place them individually in Whirlpak bags, and to measure specific conductivity.

**Table 1:** Location characteristics and standard lengths (SL) for *Poecilia reticulata* and *Poecilia picta* at sites where they formed single- or mixed-species groups.

River Characteristics					*P. reticulata*			*P. picta*		
River	Group type	Site	Coordinates	Specific conductivity (µS/cm)	n	Mean SL (mm) (SE)	Mean *Gyrodactylus* abundance (SE)	n	Mean SL (mm) (SE)	Mean *Gyrodactylus* abundance (SE)
Caroni	Single	Bend	N10 34.940 W61 16.250	0.33	41	15.4 (0.4)	2.3 (0.4)	–	–	–
		Swamp	N10 36.307 W61 25.366	62.4	–	–	–	45	19.8 (0.5)	0.5 (0.1)
	Mixed	Bridge	N10 37.037 W61 24.324	0.31	39	16.7 (0.4)	1.2 (0.3)	38	18.6 (0.4)	1.7 (0.3)
		Holstein	N10 36.527 W61 23.261	0.27	10	16.8 (1.1)	0.3 (0.2)	14	17 (0.5)	0.6 (0.3)
Guayamare	Single	Coconut	N10 34.055 W61 20.468	0.35	40	14.7 (0.4)	2.4 (1)	–	–	–
		Barrier	N10 35.548 W61 24.984	60.8	–	–	–	40	18 (0.2)	0.2 (0.1)
	Mixed	Dusty	N10 35.548 W61 24.972	0.41	29	15.9 (0.6)	0.1 (0.1)	41	18.5 (0.4)	0.5 (0.2)
		Rusty	N10 35.336 W61 24.225	0.37	40	14.2 (0.3)	0.1 (<0.1)	40	18.1 (0.2)	0.8 (0.2)
Nariva	Single	Navet	N10 20.238 W61 11.462	0.37	41	14.6 (0.4)	0.2 (0.1)	–	–	–
		Outlet	N10 24.484 W61 01.547	46.00	–	–	–	41	18.6 (0.4)	2 (0.3)
	Mixed	Poole	N10 27.822 W61 04.340	0.33	36	13.8 (0.3)	0.1 (0.1)	38	16.9 (0.4)	0.3 (0.1)

On the day of capture, each fish was anaesthetized in 0.02% Tricaine Methanesulfonate (MS-222) buffered to a neutral pH before counting all *Gyrodactylus* under a dissecting scope and taking photographs with a Nikon D80 camera for later standard length (SL) measurements to nearest 0.1 mm using ImageJ v.1.44. The fish was then euthanized in MS-222. We confirmed that the parasites were from the genus *Gyrodactylus* but were unable to identify them to the species level given the limited resolution of our field equipment.

Generalised linear mixed-effect models (GLMM) were used to test whether group type (mixed- or single-species) had an effect on whether or not an individual was infected (presence) and on the number of parasites per individual of a given species (abundance). All models included fish SL and group type as fixed effect variables, and river as a random effect variable to control for unaccounted differences among rivers. Due to variation in the difference between mixed- and single-species group types among rivers, the *P. reticulata* abundance model also included a group type within river random effect instead of a river random effect alone (i.e. the best fit model in this case included “group type|river” rather than “1|river”; χ^2^ = 20.1, df = 2, p<0.001). For *P. picta* models, specific conductivity was included as a fixed effect variable in addition to group type and SL. We used a binomial distribution of the errors with a logit link function to analyse *Gyrodactylus* presence. Abundance data was square root transformed and we used a Poisson distribution of errors with a log link function for the analysis. All analyses were performed using R Language and Environment for Statistical Computing and the lme4 package [32].

## Results

**Figure 1:**
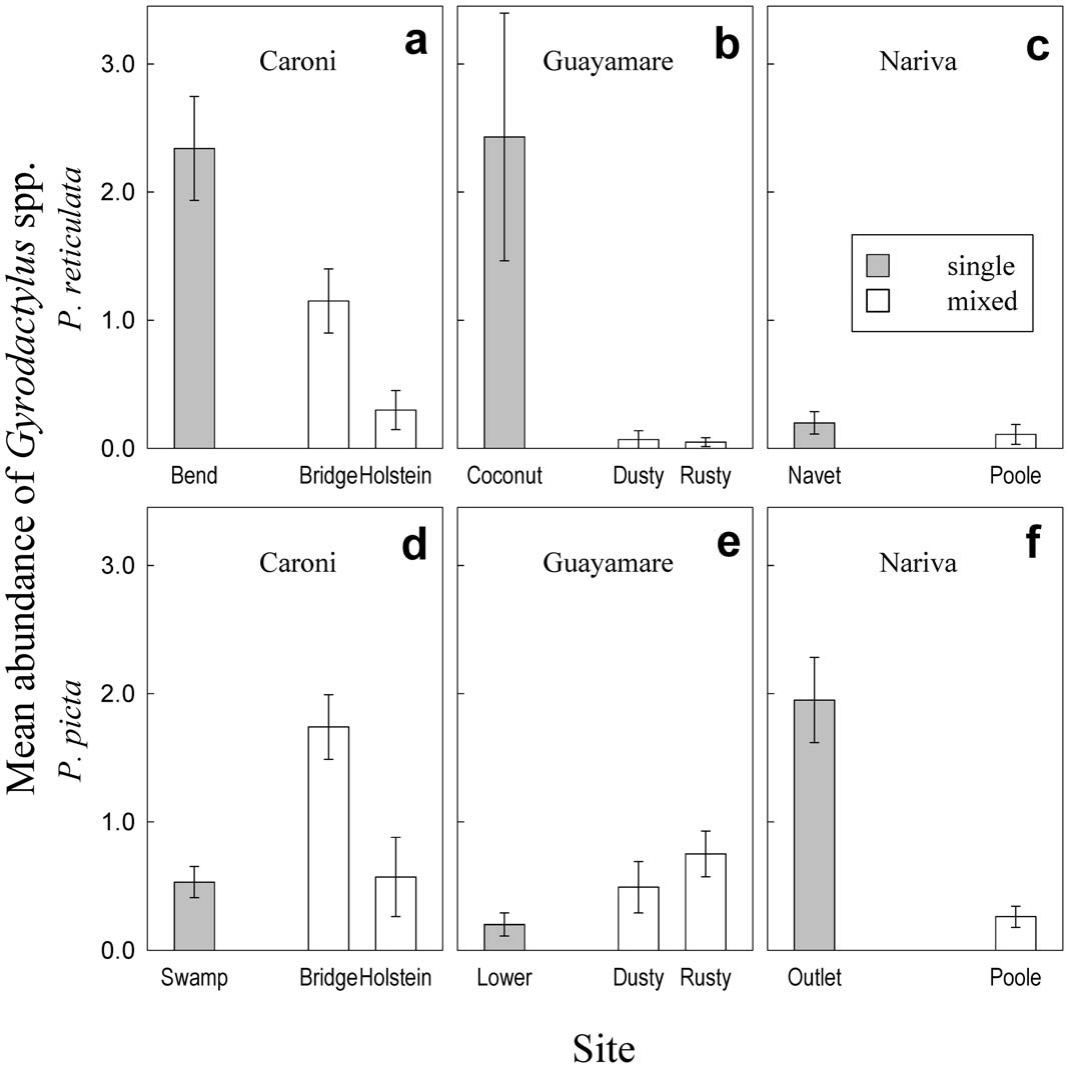
*Gyrodactylus* mean abundance. Mean abundance (+/−1 SE) of *Gyrodactylus* spp. on *P. reticulata* (**a, b, c**) and *P. picta* (**d, e, f**) in single- and mixed-species sites from three rivers in Trinidad.

Both *P. reticulata* and *P. picta* individuals were more likely to be infected in single-species groups than in mixed-species groups, and for both fish species, fish with higher SL were more likely to be infected (Table 2 - presence). For *P. picta,* the likelihood of infection decreased with increasing specific conductivity (Table 2). *Gyrodactylus* mean abundance ranged among sites from 0.05 to 2.43 parasites per fish, with average site mean abundance of 0.83 for *P. reticulata* and 0.81 for *P. picta* (Figure 1). Across single-species sites, mean *Gyrodactylus* abundance was 1.66 per fish for *P. reticulata* versus 0.89 for *P. picta*. Across mixed species sites, mean abundance was 0.34 *Gyrodactylus* per fish for *P. reticulata* versus 0.76 for *P. picta*. After accounting for the influence of SL, river and specific conductivity, the abundance of *Gyrodactylus* on individuals of both fish species was significantly higher in single-species groups than in mixed-species groups (Figure 1, Table 2). Larger *P. reticulata*, but not *P. picta*, had more parasites than smaller ones, and *P. picta* individuals at sites with higher specific conductivity had lower numbers of *Gyrodactylus* than at sites with lower specific conductivity (Table 2).

**Table 2:** Presence and abundance of *Gyrodactylus spp*. on *Poecilia reticulata* and *Poecilia picta*.

Presence									
		*Poecilia reticulata*				*Poecilia picta*			
Fixed effects		df	Estimate	SE	z-value	df	Estimate	SE	z-value
	Intercept		−4.27	1.27	−3.35***		−2.72	1.01	−2.7**
	Single	1	2.13	0.35	6.08***	1	9.44	1.01	4.59**
	SL	1	0.14	0.06	2.32*	1	0.13	0.05	2.44*
	Conductivity	–	–	–	–	1	−0.17	0.04	−4.68***
Random effects			**Std Dev**	**Corr**			**Std Dev**	**Corr**	
	River		1.3	–			0.62	–	
	Single		–	–			–	–	
**Abundance**									
		***Poecilia reticulata***				***Poecilia picta***			
Fixed effects		**df**	**Estimate**	**SE**	**z-value**	**df**	**Estimate**	**SE**	**z-value**
	Intercept		−3.21	0.82	−3.9***		−1.6	0.6	−2.65**
	Single	1	1.46	0.61	2.4*	1	6.83	1.3	5.24***
	SL	1	0.08	0.03	2.54*	1	0.05	0.03	1.67
	Conductivity	–	–	–	–	1	−0.12	0.02	−5.35***
Random effects			**Std Dev**	**Corr**			**Std Dev**	**Corr**	
	River		1.06	–			0.45	–	
	Single		0.93	−0.66			–	–	

* = p<0.05;

** = p<0.01;

*** = p<0.001.

Generalized linear mixed model results for presence (binary variable with binomial distribution) and abundance (square root transformed discrete variable with Poisson distribution) of *Gyrodactylus spp.* on *Poecilia reticulata* and *Poecilia picta*. Values given for individuals in single- relative to mixed-species groups.

**Figure 2:**
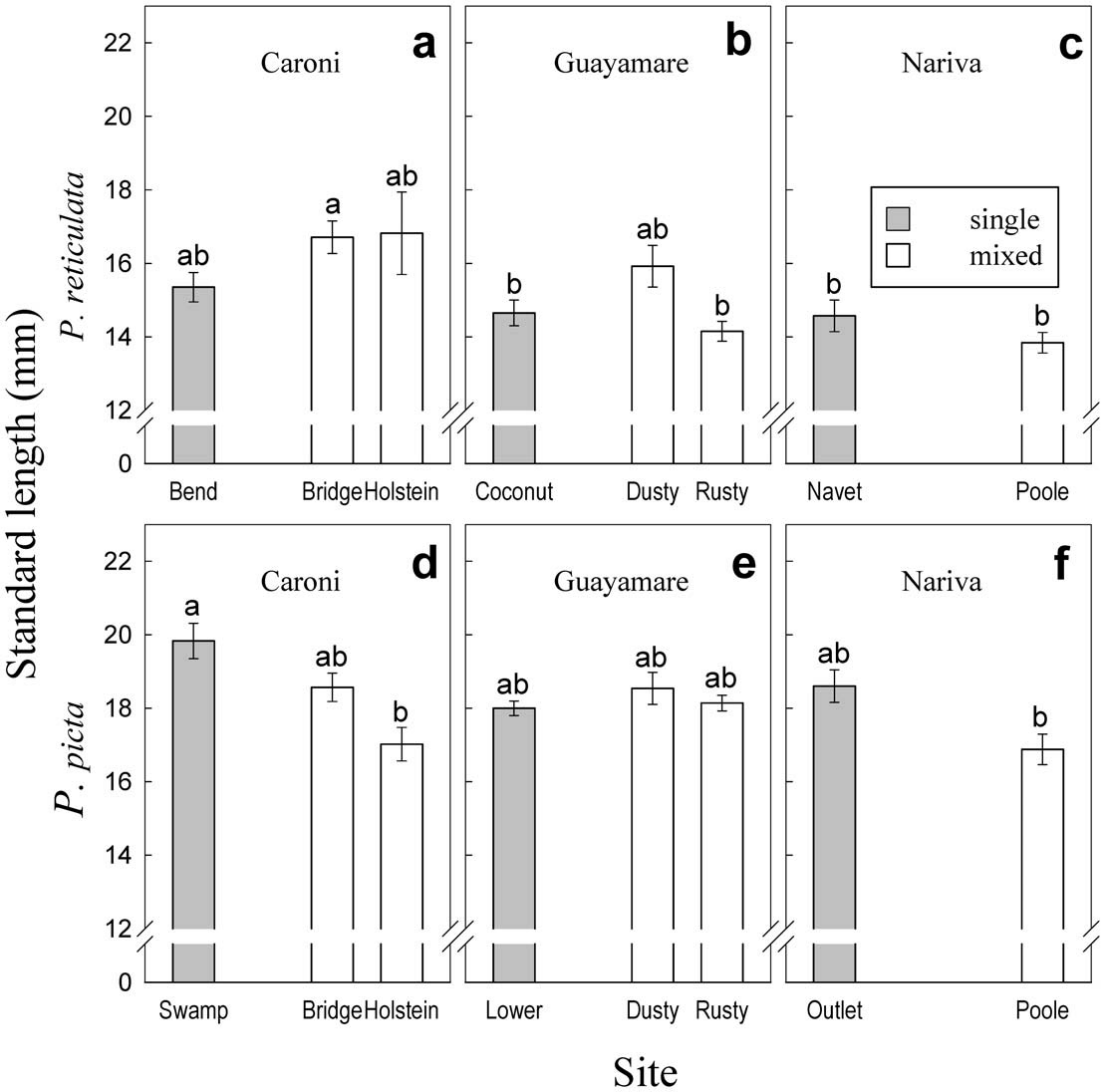
*Poecilia reticulata* and *Poecilia picta* standard length by site. Standard length (+/−1 SE) in mm of *P. reticulata* (**a, b, c**) and *P. picta* (**d, e, f**) in single and mixed-species sites from three rivers in Trinidad. Different letters denote significant pairwise differences (Tukey HSD) between sites for a given species.

The time required to catch 40 fish of both species from mixed-species sites was twice (or less) that required to catch 40 fish at single-species sites. Therefore we have no reason to presume that fish density differed between single- and mixed-species sites. We performed additional analyses to exclude the possibility that differences in presence and abundance of *Gyrodactylus* between single and mixed-species groups merely reflect size differences of fish among sites. A one-way ANOVA on the SL data with site type nested within river revealed significant size differences among rivers (F_2,267_ = 12.956, p<0.001 for *P. reticulata*; F_2,289_ = 5.097, p<0.01 for *P. picta*; Figure 2) and among site types (F_5,267_ = 3.513, p<0.005 for *P. reticulata*; F_5,289_ = 5.293, p<0.001 for *P. picta*; Figure 2). However, Tukey’s HSD post-hoc test showed that differences occurred chiefly among sites of different rivers. No significant size differences were found between single- and mixed-species sites within the same river for *P. reticulata*. *P. picta* on the other hand were significantly larger in the single-species site of the Caroni river (19.83 mm mean SL) than in one of the mixed-species sites (17.02 mm –Holstein site mean SL; Figure 2).

## Discussion

We found that *P. reticulata* and *P. picta* individuals in mixed-species groups had a lower abundance of *Gyrodactylus* and were less likely to be infected than conspecifics in single-species groups. Independent of the proximate mechanisms that determine mixed-species associations, these findings are consistent with the hypothesis that mixed-species groups provide protection for each host species from host-specific contagious parasites. However, the two fish species we studied differed in the degree of support that they provided to our hypothesis. *P. reticulata* showed clear and consistent differences between single- and mixed-species groups for both response variables (presence, abundance). For *P. picta* these differences became apparent only after controlling for specific conductivity.

In our field system, both fish species experience predation risk at all sites [33] and both species are known to be infected by host-specific contagious parasites [29], [34]. Forming groups is a common behavioural strategy to reduce the risk of predation [1] but as anti-predator benefits increase with group size, so does the risk of contagious parasite transmission. The selection imposed by these two sources of mortality will therefore affect the grouping decisions made by individuals. Forming mixed-species groups could provide an improved strategy that reduces the associated costs of contagious parasitism through a dilution effect. Contact and transmission between conspecifics is less frequent than in similarly sized single-species groups, which reduces the per-capita likelihood to contract the parasite. In addition, effective spread of the parasite through the susceptible part of the mixed-species group is probably hampered by the frequent encounter with non-susceptible hosts, a mechanism akin to the herd immunity provided to a population through vaccination [35], [36]. The dilution effect seems to be pervasive at different scales of biodiversity, from the genetic level where within population diversity increases resistance to parasites [37], [38], to the community level where higher species diversity dilutes the availability of suitable hosts [19]. The dilution effect is typically suggested for higher levels of biodiversity (e.g. [21], [39]) than our two-host-species system but the general principle of protection through relative rarity applies to our case. We suggest that the combined pressures of predation and contagious parasitism have selected for poecilid individuals that express a behavioural strategy, forming mixed-species groups, by which they reduce the costs (increased parasite burden) while maintaining the benefits (protection from predation) of joining a group. Empirical evidence relevant to our system provides support for this idea. For example, teleost fish show the ability to actively modify their grouping preferences in response to parasites [40]–[42]. More specifically, *P. reticulata* are able to discriminate individuals based on their history of infection with *Gyrodactylus* [43]–[45] and their shoaling behaviour has been shown to be heritable [46]. It is therefore likely that they can choose to preferentially associate with heterospecifics to reduce the costs of contagious parasitism.

We found that larger fish were more likely infected than smaller individuals. In addition, larger *P. reticulata* had more *Gyrodactylus*, in agreement with a previous study [47]. Larger fish provide more living space for ectoparasites [48] and size may also be associated with increased probability of contacts among hosts or the availability of energetic resources for the parasites [49]. *P. reticulata* did not significantly differ in SL within rivers although we detected a trend of slightly larger sizes at mixed-species groups (Figure 2). Therefore it is unlikely that size differences among individuals in different populations could explain the lower infections observed in mixed-species vs. single-species groups.

Our statistical models suggest that specific conductivity also played a role in the observed infection patterns. All *P. reticulata* mixed- and single-species groups occurred in the freshwater zone, thus salinity could not have driven the differences in infection between group types. However, *P. picta* single-species groups were all in sites with higher specific conductivity than mixed-species groups. Higher specific conductivity in the *P. picta* only sites could have reduced gyrodactylid survival and intrinsic rate of increase, and therefore decreased presence and abundance of the parasite. This could explain that if specific conductivity is not taken into account (Figure 1), lower abundance of *Gyrodactylus* in mixed-species sites is only evident in one river (Nariva - Figure1). Although the direct effects of salinity are unknown for *G. pictae*, Schelkle et al. [50] found that modest increases in salinity (from 0 to 12.1****µS/cm approximately) had a negative impact in *G. turnbulli* and *G. bullatarudis* growth rate and establishment on *P. reticulata*. Such effects may also occur with *G. pictae.*

A potential point of criticism is that we were not able to distinguish between the three Gyrodactylus species present in our system. This would be a point of concern regarding our results and conclusions if the parasites were generalists that can do equally well in all hosts. However, previous experiments in this system [30], [31] revealed that *G. turnbulli* and *G. bullatarudis* have faster rates of increase and their populations survive longer in *P. reticulata*.

Our study, in the light of previous experimental results and field evidence from the same host-parasite system, provides strong evidence in support of the hypothesis that individuals in mixed-species groups have lower levels of parasitism than those in single-species groups. Further research should focus on experimental studies within this system that test the effect of *Gyrodactylus* infection on host decisions to join single- or mixed-species groups and quantify the impacts of the relative proportions of the two host species on each of their *Gyrodactylus* parasite population dynamics. In addition, field studies in other systems are needed to confirm the generality of our findings. We hypothesize that as infection levels and parasite virulence increase individuals will shift preference from single- to mixed-species shoals due to the increased fitness gained through the dilution effect.
